# Association between backward walking speed and physical balance and fall risk in community-dwelling older adults

**DOI:** 10.1007/s11357-026-02149-3

**Published:** 2026-02-17

**Authors:** Ziming Gong, Cen Chen, Lefei Wang, Ryuya Tanigawa, Eisei Harayama, Sitong Li, Hiro Kishimoto

**Affiliations:** 1 Department of Behavior and Health Sciences, Graduate School of Human-Environment Studies, Kyushu University, 744 Motooka, Nishi-Ku, Fukuoka, Japan; 2 Faculty of Arts and Science, Kyushu University, IC15, 744 Motooka, Nishi-Ku, Fukuoka, Japan

**Keywords:** Backward Walking, Community-dwelling older adults, Postural control, Fall risk

## Abstract

Backward walking is increasingly recognized as an alternative task for detecting balance deficits and fall risk in older adults. However, its utility as a functional indicator in healthy populations remains underexplored. This study aimed to investigate the potential associations between the backward walking speed (BWS), physical balance, and fall risk in community-dwelling older adults. In this cross-sectional study, 90 community-dwelling older adults were recruited. Self-selected backward walking speed (SBWS) was assessed together with the Berg Balance Scale (BBS), the Performance-Oriented Mobility Assessment (POMA), and the Fall Risk Index (FRI). Grip strength and cognitive function, evaluated using the Montreal Cognitive Assessment (MoCA), were also measured as covariates. Fear of backward walking (FoBW) was investigated using a self-administered questionnaire prior to the walking tests. Pearson correlation and multiple regression analyses were performed to examine the associations between SBWS, physical balance, and fall risk. The data of 84 participants (mean ± SD age 75.7 ± 5.0 years) were analyzed. Their mean forward and backward walking speeds were 1.36 ± 0.23 m/s and 0.73 ± 0.24 m/s, respectively. The SBWS showed moderate correlations with the Timed Up and Go (TUG) test (r = –0.52), forward speed (r = 0.49), and grip strength (r = 0.41), and weaker correlations with the BBS (r = 0.38), MoCA (r = 0.23) and FES-I (r = –0.34) . No statistically significant correlations were found between SBWS and POMA-Total (r = 0.20), FRI (r = 0.09). In adjusted regression models, SBWS was significantly associated with higher BBS scores (β = 3.744, *p* = 0.023) and lower TUG times (β = –1.488, *p* = 0.029) but not with the POMA-Total (β = 1.052, *p* = 0.344) or FRI (β = 3.001, *p* = 0.096). BWS may serve as a multidimensional indicator of physical and psychological vulnerability in older adults. Longitudinal studies with dynamic or dual-task assessments are necessary to validate its predictive value for fall risk screening.

**Supplementary Information:**

The online version contains supplementary material available at https://doi.org/10.1007/s11357-026-02149-3.

## Introduction

Falls represent a major health concern among older adults, with 20–30% of falls resulting in injury, reduced mobility, and loss of independence [1]. The World Health Organization (WHO) recognizes falls as the second leading cause of accidental or unintentional injury deaths globally [1]. Epidemiological evidence indicates that most falls occur during routine daily activities, such as walking on level ground, turning, rising from a chair, or ascending/descending stairs [2]. Age-related physiological decline, particularly within systems responsible for postural control, substantially increases fall risk in older adults [3]. Impaired neuromuscular responses reduce lower limb strength, while less effective balance strategies further constrain the ability to recover from perturbations [4, 5]. Consequently, age-associated deterioration in neuromuscular, sensory, and cognitive functions diminishes the capacity to maintain postural stability and adapt to challenging walking conditions, making older adults more susceptible to instability and mobility-related difficulties [6]. Given that these tasks rely heavily on postural control, balance has been increasingly acknowledged as a fundamental element of human movement, as it plays a critical role in maintaining postural stability during both static and dynamic tasks [7]. Its deterioration can profoundly affect safety, mobility, and quality of life in older adults [8].

Given the critical role of balance in maintaining functional independence and preventing falls among older individuals, the establishment of accurate methods for assessing balance is essential in geriatric care [9]. Among the traditional and widely utilized tools for assessing balance and mobility in older adults are the Berg Balance Scale (BBS) and the Performance-Oriented Mobility Assessment (POMA) [10, 11]. These tools have been extensively validated, and numerous studies have demonstrated their strong predictive value for fall risk, underscoring their clinical relevance in identifying individuals at heightened risk of falling or otherwise losing their balance [12, 13].

Normally, forward walking is also commonly used to assess balance and functional mobility in older adults [14]. Walking is a complex motor task that requires the integration of postural control, muscle coordination, and cognitive input, making it a practical and ecologically valid indicator of balance function [15]. Parameters such as gait speed, stride length, and step variability during forward walking have been shown to reflect underlying balance impairments and predict fall risk [16]. However, recent studies suggest that backward walking may serve as an alternative tool for detecting subtle balance or mobility deficits [17]. Therefore, there is growing interest in exploring alternative gait tasks, such as backward walking, which may impose greater balance demands.

Besides, concerning the evaluation of balance, backward walking has emerged as a relatively new and promising assessment method for application in older adults, characterized by its potential for assessing both dynamic balance and mobility deficits [18]. Compared to forward walking, backward walking requires greater neuromuscular coordination, an increased cognitive demand, and more precise postural adjustments, making it a more alternative measure of balance performance [19]. Given the heightened demand that backward walking poses on sensorimotor integration and cognitive processing, it has been proposed that the measurement of individuals' backward walking speed (BWS) could be used to screen for dynamic balance and mobility deficits in independently living community-dwelling older adults [20].

Considering the limitations of forward walking tasks in detecting subtle balance impairments, particularly among high-functioning older adults, backward walking has emerged as a more demanding and alternative task for assessing dynamic balance [21]. Studies of individuals with stroke or multiple sclerosis have demonstrated excellent test–retest and inter-rater reliability for the 3-Meter Backward Walk Test (3MBWT), with an intraclass correlation coefficient (ICC) > 0.85 and minimal detectable change (MDC) values between 0.50 and 1.69 s [22, 23]. Moreover, BWS measured by the 3MBWT was shown to correlate significantly with clinical balance assessments such as the BBS and the Timed Up and Go (TUG) test [24]. In stroke populations, performance on the 3MBWT is moderately associated with BBS scores (r =  − 0.691), suggesting a link between reduced BWS and impaired dynamic balance [22]. Beyond the 3MBWT, the utility of backward walking velocity as an independent metric showed excellent reliability (ICC > 0.93) and moderate associations with prospective fall risk at 3- and 6-month follow-ups in individuals with multiple sclerosis [25].

Although several research groups have examined the relationships among backward walking, balance ability, and fall risk, several gaps remain in this field. Most existing studies have focused on specific clinical populations, such as individuals with stroke, Parkinson’s disease, or multiple sclerosis, which limits the generalizability of their findings to healthy, community-dwelling older adults. In addition, to our knowledge, none have yet integrated POMA with backward walking to assess balance. Moreover, the BBS has been reported to exhibit a ceiling effect, particularly among high-functioning older adults, reducing its ability to detect subtle balance impairments or improvements [26]. Similarly, the predictive accuracy of the POMA-Total (POMA-T) for falls has been shown to be poor, with inconsistent sensitivity and specificity across studies [27]. In addition, both the BBS and POMA are subjective assessment tools that rely on examiner observation and scoring.

The novelty of the present study lies in addressing these gaps by: (1) examining the Association between Backward Walking Speed and Physical Balance and Fall Risk in high-functioning, community-dwelling older adults; (2) investigating the association between POMA and backward walking; and (3) employing two multivariable models to evaluate the potential influence of cognitive function on these associations. (4) utilizing backward walking as an objective measurement approach to assess balance performance.

We thus conducted the present study to investigate whether BWS can serve as an alternative indicator of balance performance in community-dwelling older adults. Regarding balance, we hypothesized that faster BWS would be associated with higher BBS scores and shorter TUG times. Regarding falls, we hypothesized that faster BWS would be associated with lower Fall Risk Index (FRI) scores and higher POMA scores.

## Methods

### Study design and participants

A prior examination of the relationship between backward walking and falls indicated an effect size of f^2^ = 0.33 [28]. Based on this reference, a priori power analysis was conducted using G*Power version 3.1. The statistical test selected was a linear multiple regression (fixed model, single regression coefficient) under the t-test family with two-tails, an α level of 0.05, and a power of 0.95. The analysis estimated that a minimum sample size of 42 participants would be required to detect a similar effect size.

Considering an anticipated attrition rate of 20%, we estimated that 53 participants needed to be recruited at baseline. Based on the required sample size, a cross-sectional study was conducted by recruiting community-dwelling healthy older adults in the community of Itoshima City, Fukuoka, Japan. A total of 90 community-dwelling older adults participated in the study at Kyushu University from December 2024 to March 2025. The inclusion criteria were: (*i*) age ≥ 60 years; (*ii*) ability to follow verbal instructions; and (*iii*) ability to walk outdoors without the use of an assistive device. Individuals who self-reported neuromuscular disorders (e.g., Parkinson's disease, myasthenia gravis), central nervous system diseases (e.g., multiple sclerosis), recent injuries or surgeries that could affect gait or balance, or prior experience with backward walking training were excluded.

**Fig. 1:**
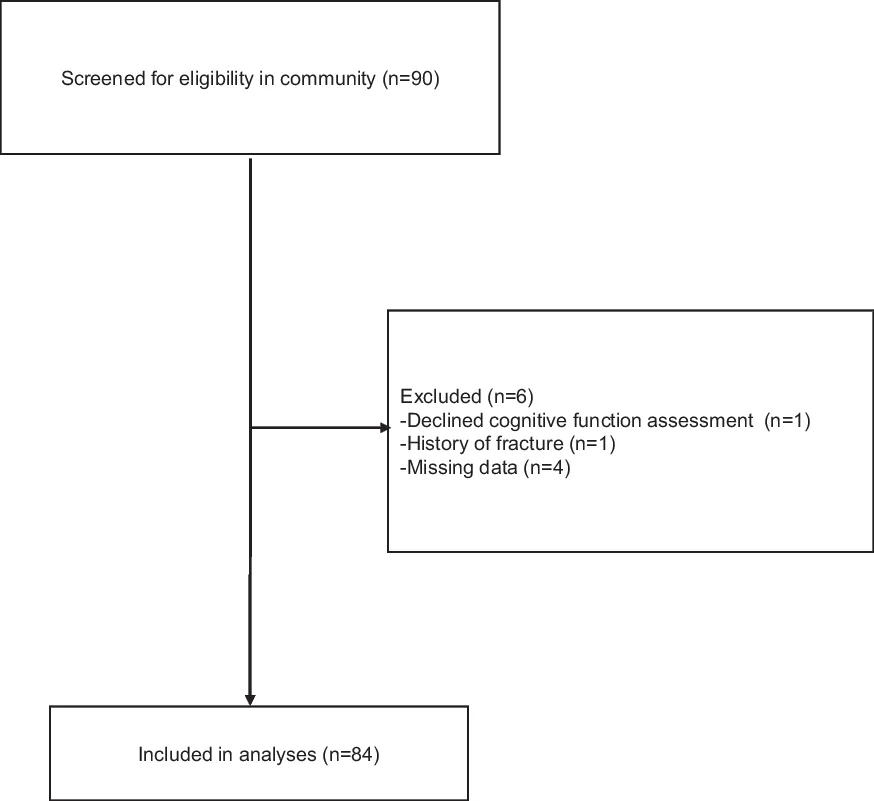
A total of 90 community-dwelling older adults (26 men and 64 women) from Itoshima City were recruited. Participants were included if they were ≥ 60 years old and able to follow verbal instructions. Individuals were excluded if they self-reported having neuromuscular disorders (e.g., Parkinson's disease, myasthenia gravis), a central nervous system condition affecting mobility (e.g., multiple sclerosis), being unable to complete the testing protocol, had any recent injuries or surgeries that could influence gait or balance, or prior experience with backward walking training

A total of 84 participants comprised the final study population. Details of the inclusion procedure are depicted in Fig. 1. This study was approved by the Ethics Committee of the Faculty of Arts and Science, Kyushu University (approval no. 202415). All of the participants provided written informed consent prior to participation and received a full explanation of the study's purpose.

### Assessments

#### Walking tests

Each of the participants completed 5.0-m forward and 5.0-m backward walking tests. They were instructed to walk barefoot on a walkway at either a "comfortable, self-selected pace" or at the "fastest possible speed within a safe range." Participants walked a total distance of 10 m for each test, including a 2.5-m acceleration zone, a 5.0-m timed zone, and a 2.5-m deceleration zone. The walking speed was recorded by a study examiner with a stopwatch, measuring the time from the moment the first toe crossed the start line to when it crossed the finish line.

Each walking condition (forward and backward) was performed twice at self-selected speeds to ensure measurement reliability. The faster of the two self-selected walking trials was used as the representative 5-m walking speed. [29, 30]. Cadence, i.e., steps/min was calculated using the following formula: Cadence (steps/min) = (number of steps/walking time [sec]) × 60.

#### Balance assessment

The participants' balance was assessed by the BBS and the TUG test. The BBS and the TUG test have been widely used to evaluate balance and to estimate the risk of falls among older adults [31, 32]. The BBS consists of 14 items with a maximum score of 56 points. A total score < 45 indicates a high risk of falls, and individuals achieving such scores as classified as having impaired balance [31]. The BBS has been reported to have excellent inter-rater reliability for assessing balance in community-dwelling older adults (ICC = 0.98) [33].

The TUG test involves standing up from a chair, walking a distance of 3 m, turning around, walking back, and sitting down in the chair again [32]. The TUG test has demonstrated good reliability in community-dwelling older adults (ICC = 0.80) [34] and excellent test–retest reliability in such populations (ICC = 0.97) [35]. For the present study's assessment, the participant was seated comfortably on a stable chair with a backrest and armrests, approximately 46 cm in height, with their arms resting naturally at the sides. The participant was then instructed to rise as quickly as possible, walk toward a fixed cone placed 3 m away, walk around the cone, return to the chair, and sit down again. The time taken to complete this sequence was recorded by the examiner. The fastest time from two trials was used for analysis.

#### Fall risk assessment

Fall risk was assessed using both simplified FRI and POMA. The FRI is a brief screening tool developed by Toba et al. for older adults living in Japanese community settings. The FRI-5 consists of the following five questions; answering 'no' was considered 0 points. (Q1) History of fall within one year; yes = 5 points. (Q2) Do you feel your walking speed declined recently?; yes = 2 points. (Q3) Do you use a cane when you walk?; yes = 2 points. (Q4) Is your back bent?; yes = 2 points. (Q5) Do you take more than five types of prescribed medicines? (polypharmacy); yes = 2 points. A cut-off score of > 6 points on the FRI-5 has been proposed to identify individuals at high risk of falling [36].

The POMA was developed by Tinetti in 1986 and is a commonly used tool for assessing mobility and fall risk in older adults. The full version (POMA-T) consists of two subscales: the balance subscale (POMA-B) and the gait subscale (POMA-G), comprising a total of 16 items, 9 related to balance and 7 to gait. The maximum score is 28 points, with 16 points allocated to balance and 12 to gait. The POMA-B evaluates an individual's balance through a series of tasks involving common movements and postural transitions encountered in daily life. The POMA-G assesses various aspects of gait patterns. On both subscales, higher scores indicate better balance and a lower risk of falling, whereas lower scores reflect impaired balance and a greater fall risk. A cut-off score < 24 points on the POMA-T has been associated with an increased risk of falls in older adults [37]. The POMA assessment typically takes approximately 10–15 min to complete and has been shown to exhibit excellent reliability and responsiveness (ICC = 0.96) [38].

### Covariates

Additional baseline information including age, sex, education level, smoking and alcohol consumption status were collected via self-administered questionnaires and structured interviews. Body mass index (BMI) was calculated as follows: BMI (kg/m^2^) = weight (kg)/height (m)^2^. We administered the Falls Efficacy Scale–International (FES-I) to assess the participants' level of concern about falling during various daily activities. A total score > 22 points was considered indicative of a high level of concern about falling [39].

Cognitive function was evaluated using the Montreal Cognitive Assessment (MoCA) developed by Ziad Nasreddine, which is designed to detect early signs of mild cognitive impairment and dementia [40]. Fujiwara et al. adapted and validated a Japanese version of the MoCA, which demonstrated good reliability with an ICC of 0.88 [41]. Fear of backward walking (FoBW) was also assessed: prior to the backward walking test the participants were asked whether they felt fearful about backward walking.

Relevant diagnoses were obtained for the participants. They were classified as having a 'diagnosis' if they had been diagnosed with any of the following conditions: hypertension, diabetes, osteoporosis, hyperlipidemia, arthritis, cardiovascular disease, hypothyroidism, ocular disease, spinal disorder, mental disorder, respiratory disease, anemia, endocrine disorder, or otorhinolaryngological disease. To assess current medication use, the participants were instructed to bring all prescription and non-prescription medications to the baseline clinic visit for verification. 'Current use' was defined as taking a given medication daily or almost daily for ≥ 30 days prior to the baseline assessment. All of the above-described variables were included in the regression models as covariates.

### Data analyses

According to a previous study, walking backward at a speed < 0.6 m/s identified 100% of fallers [19]. Therefore, baseline characteristics were compared across tertiles of backward walking speeds as follows: slow (< 0.60 m/s), moderate (0.60–0.81 m/s), and fast (> 0.81 m/s).

Descriptive data are presented as the mean (± standard deviation [SD]) for continuous variables and as the frequency (percentage) for categorical variables. The t-test for continuous data and the χ^2^-test (2 × 2) for categorical data were used to evaluate the significance of differences between the sexes. We performed a linear regression analysis to investigate the associations between backward walking speed and the physical balance and fall risk. We used two multivariable models. Model 1 was adjusted for age, gender, BMI, cadence, diagnosis, medication use, FoBW, education level, smoking, alcohol consumption, and fall efficacy. For the evaluation of the effect of cognitive function on the association, cognitive function (MoCA) was added as an additional covariate in Model 2.

We calculated the unstandardized coefficient (β) and its 95% confidence interval (CI). Pearson's correlation coefficients were calculated to examine the associations between self-selected backward walking speed (SBWS) and key outcome variables, including the balance (BBS, TUG) and fall-related measures (FRI, POMA-T). Associations with POMA subscales (POMA-B and POMA-G) were also examined. Correlation coefficients between 0.10 and 0.39 were considered to indicate a weak relationship, while those between 0.40 and 0.69 indicated a moderate relationship [42]. The data were processed using SPSS ver. 27.0 (IBM, Armonk, NY). Significance was defined as a two-tailed p-value < 0.05.

## Results

The results described in this section are mean ± SD. A total of 84 participants aged 75.7 ± 5.0 years (range 60–86 yrs) were included in the analyses: 61 women (72.6%) and 23 men (27.4%). Regarding gait parameters, the self-selected forward and backward walking speeds were 1.36 ± 0.23 m/s and 0.73 ± 0.24 m/s, respectively. In the balance assessment, the BBS score was 51.3 ± 3.1 and the TUG time was 6.60 ± 1.46 s. Regarding the fall risk assessment, the POMA-T score was 23.6 ± 2.2, and a total of 26 participants (31%) had an FRI score > 6 points, indicating an elevated risk of falling. The MoCA score for all of the participants was 23.3 ± 3.5; an exercise habit was reported by 47 participants (56%), and 33 participants (39.3%) reported FoBW prior to the test. 16 participants (19%) reported experiencing at least one fall within the past year.

Participants were divided into three groups based on their backward walking speed: the Slow (n = 28), Middle (n = 31), and Fast (n = 25) groups. The Middle group was used as the reference group in this study. Compared to the Middle group, the Slow group showed significantly higher TUG times and significantly lower self-selected forward walking speed (SFWS), grip strength, BBS, POMA-G, and MoCA scores. The Fast group demonstrated significantly higher cadence compared to the Middle group. No significant differences were observed between the Slow and Middle groups or between the Fast and Middle groups in age, gender, BMI, POMA-B, POMA-T, FES-I, diagnosis, medication use, FoBW, fall experience, FRI, smoking, alcohol consumption, years of education, exercise habit, or frailty status (Table 1).

**Table 1:** The characteristics of the participants (*n* = 84) categorized by the tertiles of their backward walking speed

	Slow SBWS < 0.60 m/s *n* = 28	Middle SBWS 0.60–0.81 m/s *n* = 31	Fast SBWS > 0.81 m/s *n* = 25
Age, yrs	77.14 ± 4.71	74.90 ± 5.51	75.04 ± 4.34
Women	25 (89.3)	20 (64.5)	16 (64.0)
SFWS, m/s	1.23 ± 0.21*	1.38 ± 0.24	1.50 ± 0.15
Cadence, steps/min	114.6 ± 15.7	109.4 ± 15.3	124.7 ± 14.0**
TUG, sec	7.60 ± 1.87**	6.28 ± 0.96	5.89 ± 0.70
Grip strength, kg	20.8 ± 4.7*	25.1 ± 6.6	26.6 ± 6.5
BMI, kg/m^2^	23.03 ± 2.89	23.36 ± 3.20	23.02 ± 3.52
BBS	49.6 ± 4.1**	52.4 ± 2.1	51.8 ± 1.8
POMA-G	9.75 ± 1.65*	10.68 ± 0.79	10.08 ± 1.08
POMA-B	13.18 ± 1.83	13.48 ± 1.46	13.56 ± 1.26
POMA-T	22.93 ± 2.81	24.16 ± 1.61	23.64 ± 1.93
MoCA, points	21.6 ± 3.5**	24.5 ± 3.0	23.6 ± 3.4
FES-I > 22	19 (67.9)	17 (54.8)	7 (28.0)
Diagnosis	26 (92.9)	26 (83.9)	23 (92.0)
Medication use	22 (78.6)	21 (67.7)	19 (76.0)
FoBW	15 (53.6)	10 (32.3)	8 (32.0)
Fall experience	4 (14.3)	8 (25.8)	4 (16.0)
FRI ≥ 6, high-risk	6 (21.4)	13 (41.9)	7 (28.0)
Smoking	0 (0)	2 (6.5)	2 (8.0)
Alcohol consumption	18 (64.3)	21 (67.7)	14 (56.0)
Education yrs < 10	3 (10.7)	4 (12.9)	2 (8.0)
Exercise habit	15 (53.6)	18 (58.1)	14 (56)
Frailty status			
Frailty	2 (7.1)	0 (0)	0 (0)
Pre-frailty	17 (60.7)	18 (58.1)	9 (36.0)
Robust	8 (28.6)	13 (41.9)	16 (64.0)

Data are mean ± SD or n (%). *p < 0.05, **p < 0.01 vs. the Middle SBWS group

*SBWS* Self-selected backward walking speed, *SFWS* Forward walking speed, *TUG* Timed Up and Go test, *BMI* Body mass index, *BBS* Berg Balance Scale, *POMA-G* Performance Oriented Mobility Assessment–Gait subscale, *POMA-B* Performance Oriented Mobility Assessment–Balance subscale, *POMA-T* Performance Oriented Mobility Assessment–Total, *MoCA* Montreal Cognitive Assessment, *FES-I* The Falls Efficacy Scale–International, *FoBW* Fear of backward walking, *FRI* Fall Risk Index

In Supplementary Table, participants were divided into two groups based on backward walking speed: the Slow SBWS group (SBWS < 0.60 m/s, n = 28) and the Fast SBWS group (SBWS ≥ 0.60 m/s, n = 56). Compared with the Fast SBWS group, the Slow SBWS group showed significantly lower self-selected forward walking speed, grip strength, BBS, and POMA-G scores, as well as significantly longer TUG times and lower MoCA scores. POMA-T scores tended to be lower in the Slow SBWS group (p = 0.050). With respect to fall-related and psychological measures, the proportion of participants with an FES-I score > 22 was significantly higher in the Slow SBWS group than in the Fast SBWS group (67.9% vs 42.9%). Frailty status also differed between groups, with frailty observed only in the Slow SBWS group. No significant differences were observed between groups in age, BMI, POMA-B, diagnosis, medication use, FoBW, smoking status, alcohol consumption, years of education, or exercise habits.

SBWS was moderately correlated with SFWS (r = 0.49, p < 0.01), TUG (r = −0.52, p < 0.01), and grip strength (r = 0.41, p < 0.01), and weakly correlated with cadence (r = 0.35, p < 0.05), BBS (r = 0.38, p < 0.01), POMA-T (r = 0.20, p = 0.075), FES-I (r =—0.34, p < 0.01), MoCA (r = 0.23, p < 0.05) and age (r = −0.24, p < 0.05). No statistically significant correlation was found between SBWS and POMA-B (r = 0.16, p = 0.156). Similarly, SFWS showed a moderate correlation with TUG (r = 0.61, p < 0.01), and weak correlations with cadence (r = 0.30, p < 0.01), grip strength (r = 0.28, p < 0.05), BBS(r = 0.26, p < 0.01), POMA-T (r = 0.22, p < 0.05), FRI (r = −0.25, p < 0.05), FES-I (r = −0.35, p < 0.01), MoCA (r = 0.19, p = 0.079) and age (r = −0.26, p < 0.05) (Table 2).

**Table 2:** Pearson's correlation coefficients for gait, balance, falls, and demographic metrics (*n* = 84)

	SBWS	SFWS	Cadence	TUG	GS	BBS	POMA-T	POMA-B	FRI	FES-I	MoCA	Age
SBWS	1											
SFWS	0.49**	1										
Cadence	0.35**	0.30**	1									
TUG	− 0.52**	− 0.61**	− 0.17	1								
GS	0.41**	0.28*	− 0.04	− 0.52**	1							
BBS	0.38**	0.26**	− 0.03	− 0.53**	0.27*	1						
POMA-T	0.20	0.22*	− 0.11	− 0.30**	0.11	0.55**	1					
POMA-B	0.16	0.13	−0.03	−0.26*	0.06	0.55**	0.84**	1				
FRI	− 0.09	− 0.25*	− 0.09	0.23*	− 0.25*	− 0.17	− 0.11	−0.07	1			
FES-I	− 0.34**	− 0.35**	− 0.10	0.30**	− 0.23*	− 0.08	− 0.12	−0.13	0.57**	1		
MoCA	0.23*	0.19	− 0.20	− 0.43**	0.29**	0.43**	0.36**	0.19	− 0.03	− 0.05	1	
Age	− 0.24*	− 0.26*	0.07	0.31**	− 0.06	− 0.35**	− 0.42**	−0.31**	− 0.01	0.06	− 0.34**	1

^*^p < 0.05, **p < 0.01

*BBS* Berg Balance Scale, *FES-I* The Falls Efficacy Scale–International, *FRI* Fall Risk Index, *GS* Grip strength, *MOCA* Montreal Cognitive Assessment, *POMA-T* Performance Oriented Mobility Assessment–Total, *POMA–B* Performance-Oriented Mobility Assessment–Balance, *SBWS* Self-selected backward walking speed, *SFWS* Self-selected forward walking speed, *TUG* Timed Up and Go test

**Fig. 2:**
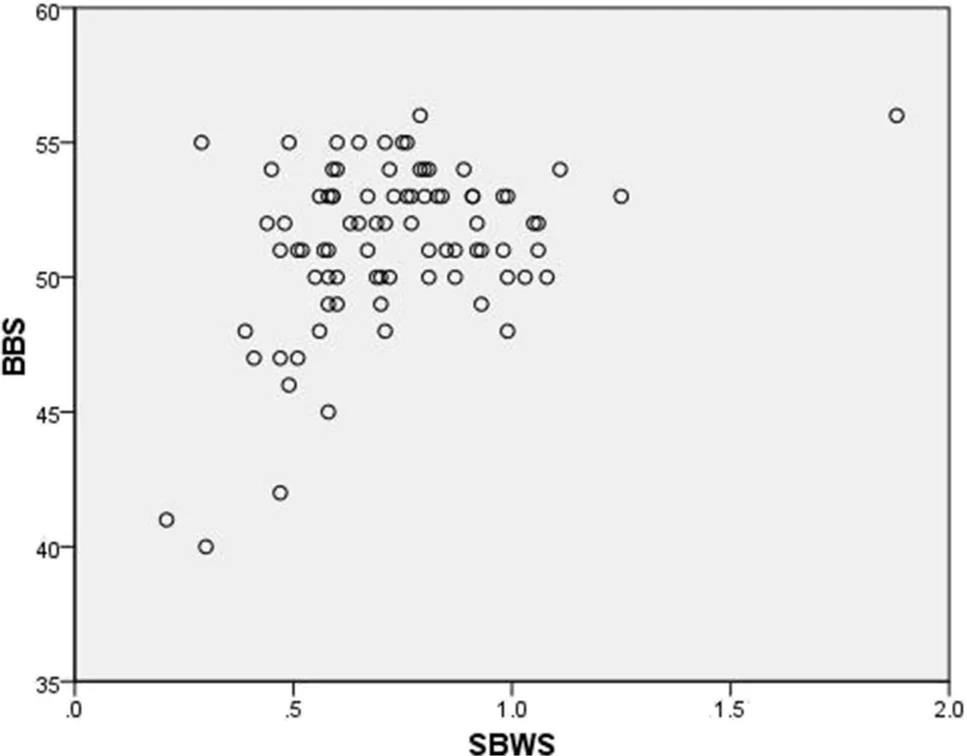
Scatter plot showing the association between self-selected backward walking speed (SBWS) and Berg Balance Scale (BBS) scores. Each circle represents an individual participant. The figure illustrates that participants with slower SBWS tended to have lower BBS scores, which may indicate reduced balance ability

In the multivariable-adjusted linear regression analysis, SBWS was positively associated with BBS scores in Model 1 (β = 4.862, 95%CI: 1.680–8.044, p = 0.003). In Model 2, which additionally adjusted for cognitive function, SBWS remained positively associated with BBS scores (β = 3.744, 95%CI: 0.536–6.953, p = 0.023) (Table 3, Fig. 2). In Model 2, the change of p-value suggests that cognitive function may be a relevant factor influencing the association between SBWS and the BBS score.

**Table 3:** The associations between balance factors (BBS and TUG) and the participants' backward walking speed (*n* = 84)

Dependent variable	Independent variable	β-value	95%CI	*p*-value
BBS	SBWS			
	Model 1	4.862	1.680 to 8.044	0.003
	Model 2	3.744	0.536 to 6.953	0.023
TUG	SBWS			
	Model 1	− 2.079	− 3.457 to − 0.737	0.003
	Model 2	− 1.488	− 2.822 to − 0.155	0.029

*BBS* Berg Balance Scale, *TUG* Turn Up and Go, *SBWS* Self-selected Backward Walking Speed, Model 1: Adjusted for age, gender, body mass index, cadence, diagnosis, medication use, fear of falling, education, smoking, alcohol consumption, fall efficacy. Model 2: Model 1 + cognitive function

**Fig. 3:**
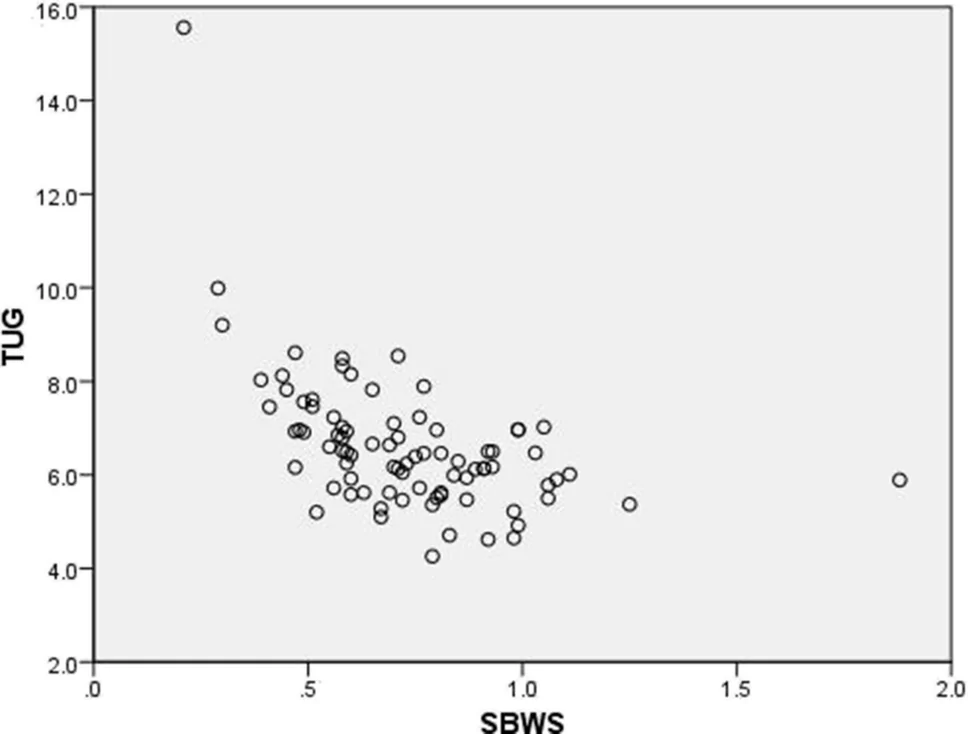
Scatter plot presenting the relationship between SBWS and Timed Up & Go (TUG) test performance. Each circle corresponds to one participant. Faster SBWS tended to be associated with shorter TUG times, which may indicate better lower-limb function and mobility efficiency

Similarly, higher SBWS was significantly associated with lower TUG scores in Model 1 (β = –2.079, 95%CI: –3.457 to –0.737, p = 0.003). In Model 2, which additionally adjusted for cognitive function, SBWS remained positively associated with TUG scores (β = –1.488, 95%CI: –2.822 to –0.155, p = 0.029) (Table 3, Fig. 3). The change in the p-value indicates that cognitive function may play an important role in the association between SBWS and functional mobility.

**Fig. 4:**
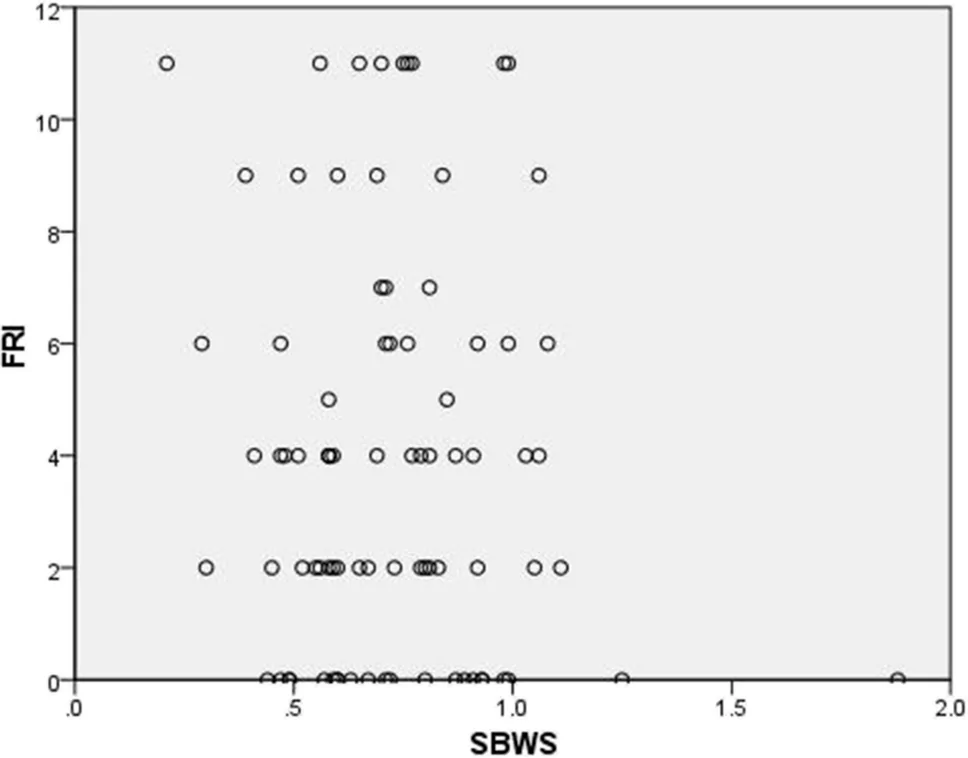
Scatter plot displaying the relationship between SBWS and Fall Risk Index (FRI-5) scores. Each point denotes one participant. No apparent relationship was found between SBWS and FRI-5

**Fig. 5:**
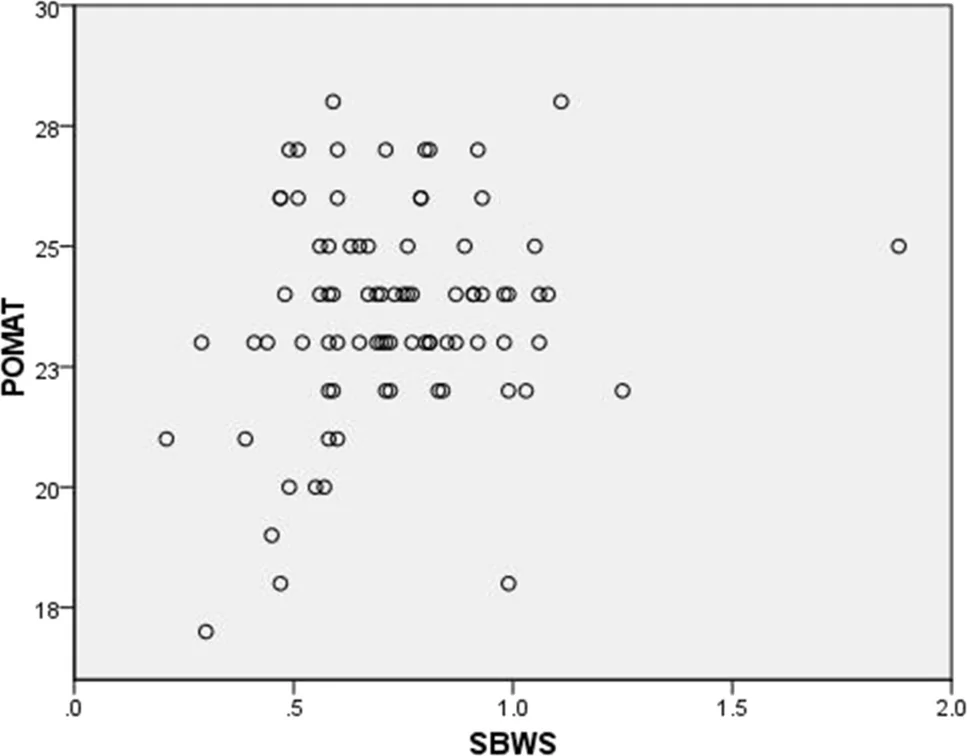
Scatter plot depicting the relationship between SBWS and total scores on the Tinetti Performance-Oriented Mobility Assessment (POMA-T). Each circle represents a participant. The association appears weak and was not statistically significant, suggesting that overall POMA performance may not strongly depend on backward walking speed in this sample

**Fig. 6:**
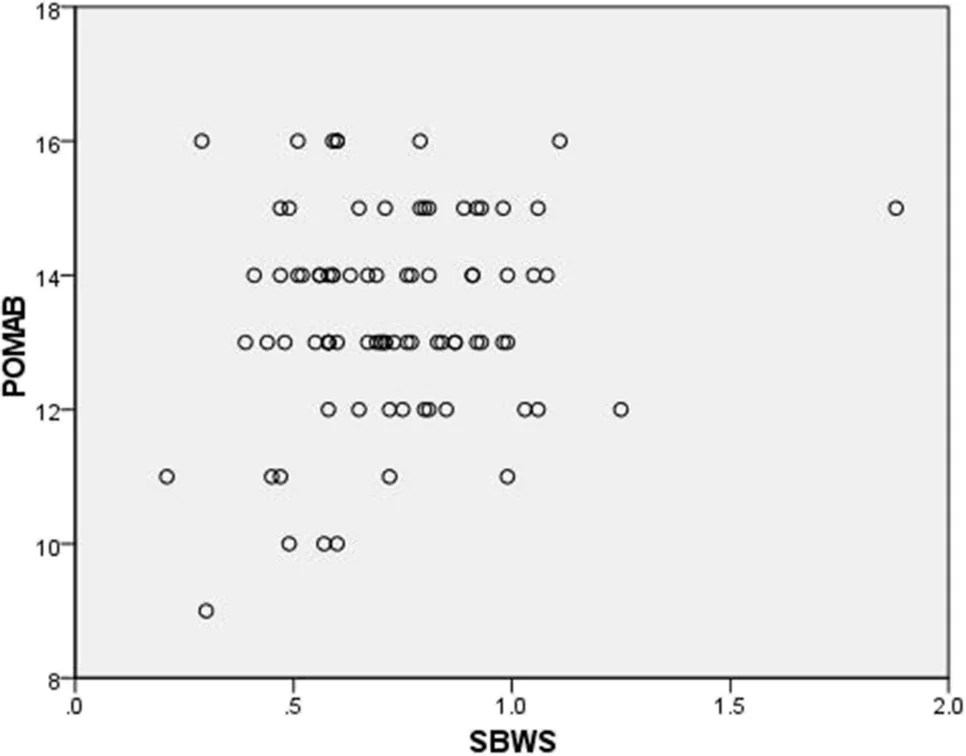
Scatter plot showing the relationship between SBWS and the balance subscale of the Tinetti Performance-Oriented Mobility Assessment (POMA-B). Each marker represents an individual participant. No distinct trend was observed

Table 4 demonstrates the association between SBWS and fall risk-related measures. No significant association was observed between SBWS and the FRI in either Model 1 (β = 2.843, 95%CI: –0.540 to 6.226, p = 0.098) or Model 2 (β = 3.001, 95%CI: –0.551 to 6.552, p = 0.096) (Table 4, Fig. 4). Similarly, SBWS was not significantly associated with POMA-T scores in either Model 1 (β = 1.052, 95%CI: –1.151 to 3.255, p = 0.344) or Model 2 (β = 0.513, 95%CI: –1.757 to 2.782, p = 0.654) (Table 4, Fig. 5). However, the change in p-value after adjusting for cognitive function in Model 2 may suggest a potential influence of cognitive function on the association between SBWS and POMA-T performance. In addition, SBWS showed no significant association with the POMA-B subscale, which specifically reflects postural balance ability, in either Model 1 (β = 0.609, 95% CI: –1.084 to 2.303, p = 0.475) or Model 2 (β = 0.478, 95% CI: –1.303 to 2.258, p = 0.594) (Table 4, Fig. 6).

**Table 4:** The association between fall risk and backward walking speed (*n* = 84)

Dependent variable	Independent variable	β-value	95%CI	*p*-value
FRI	SBWS			
	Model 1	2.843	− 0.540 to 6226	0.098
	Model 2	3.001	− 0.551 to 6.552	0.096
POMA-T	SBWS			
	Model 1	1.052	− 1.151 to 3.255	0.344
	Model 2	0.513	− 1.757 to 2.782	0.654
POMA-B	SBWS			
	Model 1	0.609	− 1.084 to 2.303	0.475
	Model 2	0.478	− 1.303 to 2.258	0.594

*FRI* Fall Risk Index, *POMAT* Performance-Oriented Mobility Assessment Total, *POMA–B* Performance-Oriented Mobility Assessment–Balance, *SBWS* Self-selected Backward Walking Speed; Model 1: Adjusted for age, gender, body mass index, cadence, diagnosis, medication use, fear of falling, education, smoking, alcohol consumption, fall efficacy. Model 2: Model 1 + cognitive function

## Discussion

BWS has been increasingly used as a clinical measure to assess balance and fall risk in older adults. This study aimed to examine whether BWS could serve as a functional indicator of balance performance among community-dwelling older adults, and our hypothesis that faster BWS would be associated with higher BBS scores and shorter TUG times was supported, aligning with other studies' findings [22–24]. However, our second hypothesis, i.e., that faster BWS would be associated with lower FRI scores and higher POMA-T scores, was not supported by our present findings.

In our study, the multiple linear regression analysis demonstrated that higher SBWS was significantly associated with higher BBS scores (β = 3.744, p = 0.023) and shorter TUG times (β =  − 1.488, *p* = 0.029) after the adjustment for confounding factors, the TUG times were significantly shorter (i.e., better) among the participants in the fastest SBWS tertile (5.89 ± 0.70 s, *p* < 0.01). These results, which are in agreement with those of prior studies, support the notion that backward walking speed may serve as an alternative indicator of dynamic balance ability [22].

Our findings are also consistent with those of Taulbee et al., who demonstrated that BWS is an alternative indicator for detecting dynamic balance and mobility deficits in community-dwelling older adults [20]. In their study, BWS showed weak correlations with the TUG results (r =  − 0.49), and in our study, the participants' SBWS showed moderate correlations with their TUG times (r =  − 0.52, *p* < 0.01). These findings further support the clinical utility of backward walking as a dynamic balance assessment [20]. Moreover, Taulbee and colleagues also highlighted that high-functioning older adults, who may score within normal limits on conventional assessments like the TUG test, can still exhibit hidden balance impairments that BWS is able to detect.

In the same study, Taulbee et al. also adopted a fall risk definition based on the guidelines from the American and British Geriatrics Societies and reported a significant association between backward walking ability and fall risk among older adults [20]. In contrast, the FRI used in the present study has relatively low sensitivity (68%) and specificity (70%) [43], which may limit its discriminatory capacity. Although the POMA was originally developed to evaluate gait and balance during forward walking, backward walking involves distinct characteristics, such as shorter step length, longer stance time, and different proportions of the stance and swing phases compared with forward walking [44]. These differences in gait performance may explain why POMA scores were not significantly associated with backward walking speed in the overall analysis. Nevertheless, the data in Table 1 demonstrate that the POMA-G scores differed significantly between the Middle and Slow BWS groups, which may suggest that backward walking speed influences gait patterns. In addition, a difference in POMA-T was also observed in the Supplementary Table, which may provide further insight for future investigations. Previous studies have highlighted the potential use of gait variability measures in augmenting the prospective evaluation of fall risk among community-dwelling older adults [15]. Furthermore, the observation that walking backward at speeds below 0.6 m/s could identify 100% of fallers [19]. According to the findings above, the observed association between POMA-G and slower backward walking speed in the present study may be partially explained. At the same time, no significant association was found between BWS and the POMA-B subscale. This finding may be explained by the fact that several tasks included in the POMA-B subscale, such as sitting balance, immediate and prolonged standing balance primarily carry the individual through positions and position changes that stress stability [11]. In contrast, backward walking is a dynamic task that requires continuous postural adjustments and coordinated movement of the lower limbs [45]. Therefore, the absence of a significant association likely reflects the different aspects of balance function captured by these two measures.

In this study, MoCA scores were positively correlated with SBWS. This finding is consistent with those of Johansson et al., who reported a significant correlation between BWS and Mini-Mental State Examination (MMSE) scores in patients with mild-to-moderate cognitive impairment due to dementia [46]. After the adjustment for the MoCA in the present study's Model 2, a potential mediation effect may exist in the associations between BWS and balance and fall risk [47, 48]. These findings suggest that cognitive function may play an important role in backward walking ability.

To better understand why backward walking is more cognitively demanding, it is important to consider its neural underpinnings. Forward walking is generally considered an automated motor task that is regulated primarily by subcortical structures such as the brainstem and spinal central pattern generators (CPGs), requiring relatively low cognitive demand. In contrast, backward walking appears to involve greater recruitment of higher-order cortical regions [49]. Neuroimaging and behavioral studies have shown that backward walking activates the prefrontal cortex, posterior parietal cortex, thalamus, and basal ganglia [49]. This may be due to the lack of direct visual input during backward movement, which forces individuals to rely on internal cognitive maps, proprioceptive feedback, and more active postural control strategies. These neural mechanisms suggest that backward walking imposes higher cognitive demands than forward walking, a difference that may be particularly pronounced in older adults and/or those with cognitive impairment.

Although the systematic research on backward walking remains limited, the existing literature indicates that gait tasks with higher cognitive demands may be more sensitive for detecting gait abnormalities in individuals with mild cognitive impairment (MCI) or early-stage dementia, potentially revealing changes earlier than conventional forward walking assessments [50]. This implies that backward walking, which poses challenges in spatial navigation and motor planning, may have potential as an early behavioral indicator of cognitive decline.

Apart from the cognitive aspects, backward walking ability has also been linked to functional and perceptual factors such as balance confidence and concern about falling. Tinetti et al. defined fear of falling as a persistent concern about falling that ultimately leads to restrictions in daily activities [51]. Previous studies have demonstrated that BWS was shown to be significantly associated with both balance ability and fall efficacy, suggesting that individuals with greater concern about falling tend to walk backward more slowly and perform more poorly on dynamic balance tasks [20]. That study also reported that FES-I scores were significantly higher in older adults who had been identified as being at risk of falling compared to those not at risk [20].

In our present investigation, FES-I scores were significantly negatively correlated with SBWS. Additionally, FES-I showed a moderate positive correlation with the FRI, indicating that the participants with greater concern about falling were more likely to demonstrate an increased fall risk. Moreover, when the participants were divided into tertiles based on their SBWS, the proportion of individuals with a high level of concern about falling was highest in the slowest SBWS group (67.9%) and lowest in the fastest group (28.0%). This further supports the potential link between backward walking ability and psychological factors related to falling.

Concern about falling can lead to compensatory gait behaviors such as reduced walking speed, shorter stride length, and prolonged double support time, as individuals attempt to increase their sense of stability [52]. However, such overly cautious gait patterns may paradoxically impair dynamic balance by reducing the adaptability and responsiveness of gait to environmental demands [53]. This effect may be particularly pronounced during backward walking, which inherently requires greater attentional control and proprioceptive input due to the absence of visual cues. Moreover, individuals with elevated concern about falling often avoid physical activities, which can lead to muscle deconditioning, impaired balance, and a subsequent increase in fall risk [54]. This behavioral avoidance further limits engagement in postural challenges, thereby weakening neuromuscular coordination and diminishing balance confidence over time. Consequently, concern about falling may not only reflect but also exacerbate deficits in postural control, particularly under demanding conditions such as backward walking.

This study examined the feasibility of using backward walking as an indicator of functional mobility. Our findings demonstrate that backward walking speed is associated not only with balance ability but also with general concern about falling and task-specific fear. Given the higher cognitive and attentional demands of backward walking, our results align with emerging neurophysiological evidence suggesting an increased involvement of higher-order cortical regions during complex gait tasks. These findings reinforce the potential of backward walking as an early behavioral marker of functional decline and suggest meaningful directions for future research in the field of geriatric medicine.

Several limitations of this study should be acknowledged. First, the cross-sectional design prevents us from drawing causal conclusions regarding the relationship between backward walking speed and balance or fall risk. Second, we examined a relatively small sample of healthy community-dwelling older adults (*n* = 84), and the findings may not be generalizable to frailer populations or individuals with neurological disorders, who may exhibit different gait characteristics. Additionally, the fall risk assessment tools used (particularly the FRI and POMA-T) may have limited sensitivity in detecting subtle impairments in high-functioning individuals. Finally, we did not assess vestibular function, which is a critical component of postural control and may influence both gait performance and balance outcomes.

## Conclusion

The results of this study demonstrated that backward walking speed is associated with balance ability in older adults. Given the cognitive and postural demands of backward walking, these findings highlight the potential of backward walking as an early behavioral marker of functional decline. However, the generalizability of our findings may be limited by the relatively small and healthy sample. Future research should address several key areas. Longitudinal studies are needed to determine the predictive value of backward walking measures over time. To improve the sensitivity of fall risk assessments in high-functioning older adults, future studies should consider incorporating more dynamic and/or dual-task-based assessment tools.

## Supplementary Information

Below is the link to the electronic supplementary material.

Supplementary file1 (DOCX 18 KB)

## Data Availability

The datasets generated and/or analyzed during the current study are not publicly available due to ethical restrictions but are available from the corresponding author on reasonable request.
