# Supplementary material for: Association between backward walking speed and physical balance and fall risk in community-dwelling older adults
Source: GeroScience. 2026 Feb 17;48(6):8531–44. doi: 10.1007/s11357-026-02149-3 (PMC13638970; doi:10.1007/s11357-026-02149-3)
Supplement: Supplementary file 1 [file 11357_2026_2149_MOESM1_ESM.docx]

Supplementary table. The characteristics of the participants (n = 84) categorized by backward walking

|  | **SBWS < 0.60 m/s**  **(n=28)** | | **SBWS ≥ 0.60 m/s**  **(n=56)** | **P-value** |
| --- | --- | --- | --- | --- |
| Age, yrs | | 77.14±4.71 | 74.96±4.98 | 0.058 |
| Women | | 25 (89.3) | 36 (64.3) | 0.015 |
| SFWS, m/s | | 1.23±0.21 | 1.43±0.21 | <0.001 |
| Cadence, steps/min | | 114.6±15.7 | 116.2±16.5 | 0.658 |
| Grip Strength, kg | | 20.8±4.7 | 25.8±6.5 | <0.001 |
| BMI, kg/m^2^ | | 23.03±2.89 | 23.21±3.32 | 0.806 |
| TUG, sec | | 7.60±1.87 | 6.11±0.87 | <0.001 |
| MoCA, points | | 21.6±3.5 | 24.1±3.2 | 0.002 |
| BBS | | 49.6±4.1 | 52.1±2.0 | <0.001 |
| POMAG | | 9.75±1.65 | 10.41±0.97 | 0.023 |
| POMAB | | 13.18±1.83 | 13.52±1.36 | 0.341 |
| POMAT | | 22.93±2.81 | 23.93±1.77 | 0.050 |
| FES-I >22 | | 19 (67.9) | 24 (42.9) | 0.031 |
| Diagnosis | | 26 (92.9) | 49 (87.5) | 0.454 |
| Medication use | | 22 (78.6) | 40 (71.4) | 0.483 |
| FoBW | | 15 (53.6) | 18 (32.1) | 0.058 |
| Smoking status | | 0 (0) | 4 (7.1) | 0.147 |
| Alcohol consumption | | 18 (64.3) | 35 (62.5) | 0.873 |
| Education year < 10, n (%) | | 3 (12.0) | 6 (11.1) | 1.000 |
| Exercise habit | | 15 (53.6) | 32 (57.1) | 0.756 |
| Frailty status | |  |  |  |
| Frailty | | 2 (7.1) | 0 (0) | 0.043 |
| Pre-frailty | | 17 (60.7) | 27 (48.2) | 0.280 |
| Robust | | 9 (32.1) | 29 (51.8) | 0.053 |

Data shows mean ± standard deviation or n (%).

SBWS: self-selected backward walking speed, SFWS: self-selected forward walking speed, BMI: body mass index, TUG: Timed Up and Go test, MoCA: Montreal Cognitive Assessment, BBS: Berg Balance Scale, FRI: Fall Risk Index, POMA-G: Performance Oriented Mobility Assessment–Gait subscale, POMA-B: Performance Oriented Mobility Assessment–Balance subscale, POMA-T: Performance Oriented Mobility Assessment–Total, FES-I: The Falls Efficacy Scale–International, FoBW: fear of backward walking
